# Testosterone Levels, Challenging Parenting Behavior, and Protective Parenting Behavior: A Correlational Study Among First‐Time Fathers in the First Year of Fatherhood

**DOI:** 10.1002/dev.70160

**Published:** 2026-05-08

**Authors:** Annemieke M. Witte, Carolina Santos, Mirjana Majdandžić, Marinus H. van IJzendoorn, Marian J. Bakermans‐Kranenburg

**Affiliations:** ^1^ Clinical Child & Family Studies VU Amsterdam Amsterdam The Netherlands; ^2^ William James Center for Research Ispa–Instituto Universitário Lisboa Portugal; ^3^ Research Institute Child Development and Education University of Amsterdam Amsterdam The Netherlands; ^4^ Facultad de Psicología y Humanidades Universidad San Sebastián Valdivia Chile; ^5^ Department of Psychiatry Monash University Melbourne Australia; ^6^ Research Department of Clinical, Educational and Health Psychology University College London London UK

**Keywords:** activation parenting, infancy, observations, protection, sensitive parenting

## Abstract

Only few studies have examined whether testosterone levels are involved in parenting behaviors other than sensitivity and nurturing behavior. This preregistered study examined associations between fathers’ hair testosterone levels, challenging parenting behavior (CPB), and protective parenting. Seventy first‐time partnered fathers with a healthy‐born infant (*M* = 6.70 months, SD = 2.16) visited our research center for hormonal and behavioral assessments. Hair samples were collected to measure testosterone concentrations over the past month. CPB was observed during father–infant free play interaction and coded for the extent to which fathers verbally and physically challenged their infants to act outside their comfort zone. Paternal sensitivity was independently rated and analyzed exploratively. Protective parenting behavior was observed during the auditory startling task, which involved exposing the dyads to a short, unexpected loud sound. No significant correlations between fathers’ hair testosterone levels, CPB, and protective parenting emerged. Exploratory analyses (not preregistered) showed that fathers with lower testosterone levels were more sensitive. Future studies with larger and more diverse samples may benefit from incorporating multiple testosterone indices (e.g., hair, diurnal, and reactivity measures) to examine whether these indices are differentially related to CPB and protective parenting.

## 1 Introduction

Meta‐analytic and systematic reviews have directed attention to differences between maternal and paternal parenting styles as well as father's unique contributions to their children's development (Jeynes 2016; Yaffe 2023). Fathers typically engage in more physical and playful interactions with their children than mothers (Cabrera et al. 2000; Paquette 2004; St George and Freeman 2017). Building on this perspective, Majdandžić et al. (2016) developed the construct of challenging parenting behavior (CPB) and an accompanying questionnaire and observation method to assess it. CPB refers to the extent to which parents, physically and verbally, encourage their children to move outside their comfort zone and push their limits. CPB behaviors include, for example, rough‐and‐tumble play, lifting the infant in the air, encouraging infants to stand by gently pulling them up by the arms, approaching the infant with growling noises, and making arousing sounds (Majdandžić et al. 2016). Although both mothers and fathers may show CPB, it tends to be considered as more typical for fathers (Paquette 2004).

Around the same time as the growing attention to CPB, others drew attention to the importance of parental protection (Bakermans‐Kranenburg and Van IJzendoorn 2017) and developed observational methods to assess protective behavior in semi‐naturalistic laboratory settings (Lotz et al. 2021). Parental protective behavior refers to any behavior that safeguards children from (potentially) risky, dangerous, and harmful situations. To date, few studies have examined hormonal associations with CPB or protective behavior, even though hormones have been linked to various other parenting behaviors (e.g., Bakermans‐Kranenburg et al. 2019; Rilling 2013). The aim of this preregistered study is to examine whether and how father's testosterone levels measured in hair are associated with CPB and protective behavior. We focus on first‐time fathers with a single healthy born infant between 2 and 12 months old. Results of this study will deepen our understanding of how hormonal processes are related to fathering behaviors.

### 1.1 Testosterone Levels and Sensitive Parenting

The hormone testosterone plays a significant role in the development and maintenance of typical male physical characteristics such as muscle growth, body hair, and a lower tone of voice (Zitzmann and Nieschlag 2001). Testosterone is also known for its associations with competitive and aggressive behavior. Testosterone levels rise in anticipation of competition (Cheng and Kornienko 2020) and male winners were found to have higher testosterone levels compared to male losers (*d* = 0.23, *k* = 44; Geniole et al. 2017). Meta‐analytic evidence showed that male testosterone levels were positively associated with aggressive behavior, although associations were weak (*r*  =  0.07, *k* = 87) (Geniole et al. 2020). Such associations might be relevant for parenting behavior as well.

In the context of parenting, meta‐analyses found that fathers have lower salivary (or sometimes blood) testosterone levels than men without children, although the combined effects sizes indicated relatively modest effects (*r* = 0.19, *k* = 22 in Grebe et al. 2019; *r* = 0.11, *k* = 20 in Meijer et al. 2019). Additionally, Meijer et al. (2019) reported that fathers with lower testosterone levels showed higher parenting quality, but the reported effect size was small (*r* = 0.07, *k* = 18). For example, some studies showed that lower paternal salivary testosterone levels were associated with higher levels of involvement (Kuo et al. 2018), the provision of more direct care (Alvergne et al. 2009), more sympathy for a crying infant (Fleming et al. 2002), and longer and more frequent speech directed at the child (Weisman et al. 2014). These findings are in line with the steroid/peptide theory of social bonds (van Anders et al. 2011), which proposes a framework for how testosterone (in addition to oxytocin and vasopressin) levels contribute to parenting behavior, with the idea that lower testosterone levels are linked to more nurturing and affiliative parenting behaviors. However, it is unclear whether the theory's proposed associations emerge only when diurnal or reactivity hormonal measures are used or also when longer term and more stable hormonal measures are used.

Of note, the association between fathers’ testosterone levels and parenting behavior is complex: reciprocal associations have been reported (e.g., De Vries et al. 2019; Gettler et al. 2011), along with nonsignificant associations between fathers’ testosterone levels and paternal sensitivity (Bakermans‐Kranenburg et al. 2022; Bos et al. 2018; Endendijk et al. 2016; Lotz et al. 2022). In some studies testosterone interacted with other hormones to predict insensitive parenting (Bakermans‐Kranenburg et al. 2022; Bos et al. 2018) or showed only associations with sensitivity when diurnal patterns or reactivity levels were considered (De Vries et al. 2019; Endendijk et al. 2016). Taken together, while most studies on the association between testosterone and parenting behavior have focused on the link between fathers’ salivary testosterone levels and sensitive parenting behaviors, the findings are mixed. Meta‐analyses suggest that higher salivary testosterone is modestly related to less sensitive parenting, but it is unknown whether that result holds (or is stronger) for hair testosterone.

### 1.2 Testosterone Levels and CPB

The construct of CPB was developed following Paquette's (2004) proposition that when fathers excite, surprise, and momentarily destabilize the child within a safe and secure context, they help their children to become more open to the world, confident, and resilient. In line with this reasoning, studies have found that fathers with more CPB (both observed and self‐reported) had infants and children who displayed less (social) anxiety symptoms (Lazarus et al. 2016; Majdandžić et al. 2014, 2018; Möller et al. 2015).

Of interest to the present study is the finding that fathers who engaged in higher levels of rough and tumble play with their 1‐ to 5‐year‐old children showed a steeper decline in their diurnal salivary testosterone levels (Ahnert et al. 2021). No studies have explicitly examined associations between fathers’ salivary testosterone levels and activating parenting behaviors, such as CPB. However, relevant research has found that higher testosterone levels are related to more sensation‐seeking behaviors (Apicella et al. 2015; Stanton et al. 2011). In one study, fathers with low testosterone levels reported the lowest levels of sensation seeking behaviors at 2 months postpartum (Perini et al. 2012). A later study replicated this association (when controlling for the D4 dopamine receptor) (Campbell et al. 2010). In conclusion, these findings suggest that fathers with higher testosterone levels may be more inclined to exhibit the activating, exciting and boundary‐pushing behaviors central to CPB. However, this suggestion remains speculative due to the limited evidence available.

### 1.3 Testosterone and Protective Behavior

Parents’ protective behavior is of critical importance to guarantee children's survival and to safeguard them from illness, injury, and external threats (Bakermans‐Kranenburg and Van IJzendoorn 2017). However, studies that have examined parental protective behavior are scarce, which is understandable because ethically acceptable methods to assess protective behavior in real‐life settings are limited (Bakermans‐Kranenburg and Van IJzendoorn 2017). The steroid/peptide theory of social bond's proposes a link between lower testosterone levels and more nurturing and affiliative parenting behaviors and in addition suggests that higher testosterone levels are associated with more defensive or protective parenting responses, probably because higher testosterone levels prepare the body for action in threatening situations (van Anders et al. 2011). Indeed, men's testosterone levels increased when they listened to infant cry sounds and could not provide a nurturing response but decreased when they could provide a nurturing response (van Anders et al. 2012). Furthermore, after testosterone administration, men displayed increased neural activity in brain areas associated with threat processing when viewing angry faces (Goetz et al. 2014). However, in a sample of fathers with infants between 2 and 5 months old, no associations were found between testosterone levels and self‐reported protective behavior, observations of protective behaviors, or neural responses to infant threatening situations (Lotz et al. 2021). Overall, little is known about how fathers’ testosterone levels relate to observed protective parenting behavior, but theories suggest that testosterone is associated with more protective behavior in threat contexts.

### 1.4 The Current Study

One major difference between this study and the above‐described literature is that we measured testosterone from hair samples rather than saliva samples (e.g., Lotz et al. 2021; Weisman et al. 2014; van Anders et al. 2011). Hair samples reflect cumulative secretion of testosterone over the preceding months (with each centimeter of hair corresponding to approximately 1 month of secretion). Consequently, hair samples provide a more stable measure than saliva or blood samples, which are influenced by circadian rhythms and external factors (Ferrari et al. 2025). Prior studies have reported moderate associations between testosterone levels in hair and saliva (Chen et al. 2019; Stern et al. 2022).

The aim of this preregistered study is to examine whether fathers’ hair testosterone levels are associated with CPB and protective parenting in infancy. Infancy is a developmental period in which parental protection is crucial for safety and survival (Bakermans‐Kranenburg and Van IJzendoorn 2017). At the same time, this period is characterized by playful parent–child interactions (R. Feldman 2023; J. Feldman and Shaw 2021). This makes infancy a particularly important period for studying these parenting behaviors.

We examined whether the previously reported associations between testosterone levels and paternal behavior are specific to diurnal patterns and reactivity measures or also emerge when longer term and more stable hair testosterone measures are used. We hypothesized, albeit cautiously given that studies using hair testosterone are scarce, that fathers with higher hair testosterone levels would show more CPB and more protective parenting behaviors. Additionally, we hypothesized that higher levels of CPB are associated with less protective parenting behaviors. Although the combination of the first and second hypothesis may seem counterintuitive, conceptually they are not in contrast with each other. Testosterone has been associated with activation‐related behaviors that may manifest differently depending on the context. In safe and playful situations, higher testosterone levels may relate to CPB, whereas in potentially threatening situations, higher testosterone levels may relate to protection. The two behaviors themselves may still show a negative association, as fathers who actively encourage their child to take on challenges and tackle risks may be less inclined to show protective responses, and vice versa.

## 2 Method

### 2.1 Procedure

This research is part of *Fathers Today*, a randomized controlled trial (RCT) investigating the effects of hormonal administration on fathering outcomes (Witte et al. 2019). Fathers were invited for three research visits during which they self‐administered a nasal spray containing oxytocin, vasopressin, or placebo, with order randomized and 1‐ to 2‐week intervals between administrations. For this study, data from the placebo condition were used to assess CPB. Data from the second research visit were used to assess protective parenting behavior, as this data were only collected during this timepoint to avoid potential learning effects. At the start of the first research visit, hair strands were collected to assess testosterone levels. Fathers reported on potential confounders for testosterone level via a questionnaire (Rippe et al. 2016) during the first research visit. Following data collection in 2020–2021, the study was preregistered in 2025, after which the statistical analyses were conducted in accordance with the preregistration. The preregistration can be found on the Open Science Framework (OSF; https://osf.io/pmh6x/overview). We deviated from our preregistration in one way, that is, we preregistered the expectation that higher testosterone levels would be associated with *less* protective parenting behavior. However, while drafting the introduction and prior to conducting any data analysis, we realized that the arguments to expect a positive association between testosterone and protective parenting were stronger.

### 2.2 Participants

The sample included 70 first‐time fathers between 22 and 49 years of age (*M* = 35.56, SD = 4.60) with a healthy infant of on average 6.70 months of age (SD = 2.16, range 2–12 months). Infants (54% boys) were born full‐term (after 37 weeks of gestation) and from singleton pregnancies. Fathers were fluent in the Dutch language and resided in the same household as the infant and mother. All but one father was born in the Netherlands. Fathers’ educational level was high: 67% had a university master's degree, 24% had a university bachelor's degree, and 9% had completed secondary vocational education. Participants were recruited via the municipality of Amsterdam in the Netherlands. The municipality sent letters to all fathers in Amsterdam who were registered at the same address as their partner (the mother) and their infant under the age of 1 year. Fathers indicated their willingness to participate by returning the attached response card. Fathers subsequently received another letter containing detailed information about the study. As this study was part of a larger RCT, exclusion criteria included factors that interfere with hormonal measures and fMRI assessment. Fathers were excluded in case of neurological disorders, endocrine diseases, psychiatric disorders, cardiovascular diseases, use of psychoactive medications, nose injuries and disorders, magnetic resonance imaging contraindications, regular use of soft drugs, hard drug use within the past 3 months, or excessive alcohol intake. Both parents needed to have parental authority. A researcher verified inclusion and exclusion criteria during a telephone call.

The study was approved by the ethics committee of the Leiden University Medical Center (LUMC; protocol NL70143.058.19) and registered in the Overview of Medical Research in the Netherlands (OMON). The study protocol was published prior to the start of the study (Witte et al. 2019). Prior to data collection, fathers provided written informed consent for their own participation, and both mothers and fathers provided written informed consent for their infant's participation. Fathers’ travel expenses were reimbursed, and they received financial compensation for their participation in each phase of the study, up to a maximum of €130. After completion of all visits, the infant received a small present.

### 2.3 Instruments

#### 2.3.1 Testosterone Levels

Father's testosterone levels were assessed using hair strands approximately 3–5 mm in diameter and 3 cm in length. Hair samples were cut by the first author or a research assistant from the center of the back of the head, as close to the scalp as possible, near the inion of the occipital protuberance. Samples were wrapped in tin foil packages and kept at room temperature. Once all samples had been collected, they were shipped to Dresden Labservices GmbH (http://www.labservice‐dresden.de) for analysis. The 1 cm hair segment closest to the scalp was assessed for testosterone concentrations using liquid chromatography‐tandem mass spectrometry with online solid phase extraction. Given that hair typically grows at a rate of 1 cm per month, this 1 cm segment reflects testosterone secretion in the month prior to hair sampling (Stalder et al. 2017). Inter‐ and intra‐assay coefficients of variation for hair testosterone concentrations were < 10% in comparable samples analyzed in the same lab (Gao et al. 2013). To assess reliability, 36% of the hair samples were randomly selected for duplicate analysis. The average intra‐assay coefficient of variation was 6%. For hair segments that were assayed in duplicate, the average score of the two values were used in the analyses. Due to positive skewness, a log transformation was applied to approximate a normal distribution.

#### 2.3.2 Challenging Parenting Behavior

CPB was observed during a 10‐min videotaped free play session in the placebo condition, which began with 5 min of play *without* toys and was followed by 5 min of play *with* toys. Fathers were asked to play with their infant in their usual way. CPB was assessed through two scales, a *verbal* and *physical* scale. The *verbal* scale focuses on the use of language (e.g., 1, 2, 3…yes stand up!) and vocalizations (e.g., playful grunting and growling). The *physical* scale focuses on actions such as touching, lifting, swinging, or throwing the infant wildly in the air (Majdandžić et al. 2016). Scores were assigned for (1) *physical* CPB during free play *without* toys, (2) *verbal* CPB during free play *without* toys, (3) *physical* CPB during free play *with* toys, and (4) *verbal* CPB during free play *with* toys. For each 1‐min interval, coders used a 5‐point rating scale to rate physical and verbal CPB, with 1 = *no CPB*, 2 = *slight CPB*, 3 = *somewhat CPB*, 4 = *clear CPB*, and 5 = *very clear CPB*. Both the frequency and intensity of the behavior were considered in the scoring. For example, throwing the infant once high into the air could receive a similar score as more frequent and moderately challenging behaviors. Coders also considered the child's developmental age, meaning that behaviors such as encouraging the infant to sit were rated as less challenging if the child already mastered this skill. Minute‐by‐minute scores were averaged into one global score for each of the four observational contexts. Strong correlations were found between *physical* and *verbal* CPB *without* toys (*r* = 0.57, *p* < 0.001) and between *physical* and *verbal* CPB *with* toys (*r* = 0.70, *p* < 0.001). Therefore, task scores for CPB *without* toys and for CPB *with* toys were created by averaging the *physical* and *verbal* subscale scores. Higher scores reflect higher levels of CPB. Coding was performed by five advanced bachelor and master students in child and family sciences. The coders received training from A.W., who was herself trained and supervised by M.M. (the expert coder). The average ICC (single measures, absolute agreement) between the first author and trained coders was 0.66 for physical CPB *without* toys, 0.81 for verbal CPB *without* toys, 0.70 for physical CPB *with* toys, and 0.83 for verbal CPB *with* toys. Coders were blind to placebo condition assignment. CPB data were collected during the placebo condition at the first visit (*n* = 21 fathers), the second visit (*n* = 21 fathers), or the third visit (*n* = 19 fathers).

#### 2.3.3 Protective Parenting Behavior

Protective parenting behavior was coded from the videotaped auditory startling task (AST) (Lotz et al., 2021; Riem et al. 2023). In the second research visit, the AST followed the 10‐min free‐play interaction. During the AST, fathers and infants are exposed to a short, unexpected loud sound from a hidden audio installation. The sound consisted of white noise (80 dB) and was set to play for 10 s with brief pauses. Once the loud sound ended, the researcher entered the room and offered an apology. Researchers explained that the sound was caused by a technical issue. Upon completion of the third research visits, fathers were debriefed about the AST. Scores were assigned on a 10‐point rating scale, with 1 = *least protective* and 10 = *most protective*. A score of 1 was given when the father covered his own ears and showed no attention to the infant, a score of 4 was given when the father looked at the infant and tried to distract the infant (e.g., with toys), and a score of 10 was given when the father protected the infant by covering the infant's ears within 5 s of noise onset. Higher scores reflect more protective behavior. Videos were coded by A.W. and one master student in child and family sciences, who was trained by A.W. Coders were blind to condition assignment. The average ICC (single measures, absolute agreement) between A.W. and the trained coder was 0.96 for protective behavior. When various protective responses were observed, the highest score was assigned. Protective parenting behavior data for four fathers were collected during the third research visit (instead of the second) because of technical issues with the AST.

#### 2.3.4 Paternal Sensitivity

Paternal sensitivity was included for post hoc exploratory analyses. It was observed during the 10‐min videotaped free play session in the placebo condition and included 5 min of play *without* toys and 5 min of play *with* toys (i.e., similar to CPB). Coding was performed using the Ainsworth scales for sensitivity and cooperation (Ainsworth et al. 1974). The sensitivity scale focuses on parents’ ability to accurately perceive infant signals, while responding to them promptly and accurately. The cooperation scale focuses on the extent to which parents physically cooperate with the child and avoid interfering with the infant's ongoing activity. Scores ranged from 1 = *highly insensitive or highly interfering* to 9 = *highly sensitive or highly cooperative*. The expert coder (M.B.K.) trained three coders (one postdoctoral researcher, one advanced bachelor's student in psychobiology, and one master's student in child and family sciences). The average ICC (single measures, absolute agreement) between the expert coder and trained observers was 0.75 for sensitivity and 0.78 for cooperation. Scores for sensitivity and cooperation were highly correlated (*r*(60) = 0.65, *p* < 0.001) and averaged into a single score. Coders were blind to placebo condition assignment. Different coders coded paternal sensitivity and CPB.

#### 2.3.5 Covariates

Fathers self‐reported on potential confounders using a questionnaire that was based on the Confounders in the Measurement of Corticosteroids in Hair (CoMCoH) questionnaire (Rippe et al. 2016), which was previously used by Verhees et al. (2021). The following questions were included: “How often did you wash your hair per week in the past months” (recoded as: ≤ three times per week vs. > three times per week), “When was the last time you washed your hair” (recoded as: within 24 h before sampling—yes or no), “Is your hair currently dyed” (yes/no), “How many times per week did you use hair products (e.g., hair gel) in the past months” (recoded as: ≤ three times per week vs. >three times per week), and “When was the last time you used a hair product” (recoded as: within 24 h before sampling—yes or no). Moreover, participants reported on their length and weight to calculate BMI, and their highest completed education level as an indicator for socioeconomic status. Astronomical season was based on the date of the research visit. Finally, hair segments were weighed at Dresden Labservices GMBH to control for hair mass. Because none of the participants had dyed their hair, the covariate “hair dyed” was not used in any analyses. A univariate ANOVA based on complete cases showed that fathers who had washed their hair in the past 24 h had lower hair testosterone levels compared to fathers who had not, *F*(1,41) = 9.70, *p* = 0.003. Therefore, hair testosterone values were corrected for hair washed in the past 24 h via residualizing. Fathers’ hair testosterone levels were not associated with hair washing frequency, hair product use frequency, timing of hair product usage, astronomical season, hair weight segment, BMI, and educational level. Furthermore, fathers’ hair testosterone levels were not associated with father's age, child sex, child age, and fathers’ self‐reported depressive symptoms as measured after the first research visit with the Edinburgh Postnatal Depression Scale (EPDS; Cox et al. 1987).

### 2.4 Missing Data

Rates of missing data per variable were 38.6% for hair testosterone levels, 12.9% for CPB, 21.4% for protective parenting behavior, and 14.3% for paternal sensitivity. Little's MCAR test was not significant (*χ* ^2^(15) = 20.51, *p* = 0.153), indicating that data were missing completely at random. Reasons for missing data are depicted in Figure S1.

### 2.5 Power Analysis

A sensitivity power analysis conducted in G*Power with a correlational bivariate normal model, a sample size of *n* = 70, a power level of 0.80, an alpha level of 0.05, and a two‐tailed test (given the exploratory nature of the study) showed that the study was sufficiently powered to detect effect sizes of *r* = 0.32. This effect size can be interpreted as medium according to Cohen (1988), and as relatively large based on the more recent guidelines proposed by Gignac and Szodorai (2016). G*power does not include a power analysis for partial correlational analyses. Therefore, we performed a sensitivity power analysis for a linear multiple regression (fixed model, *R* ^2^ increase) as this model allows for the inclusion of covariates. With a sample size of *n* = 70, a power level of 0.80, an alpha level of 0.05, one tested predictor, and a total number of two predictors (one covariate; experimental condition), we had sufficient power to detect an effect size of *f* ^2^ = 0.12, which corresponds roughly to *r* ≈ 0.33.

### 2.6 Data Analysis Plan

Missing data were imputed through predictive mean matching using the MICE (Multivariate Imputation by Chained Equations) package (Van Buuren and Groothuis‐Oudshoorn 2011) in R. Fifty imputed datasets were generated with 20 iterations per imputation. The imputed datasets were subsequently combined into a single dataset using the *merge imputations* function of the *sjmisc* package. This function calculates the mean of the imputed values across all datasets (*m* = 50) for each missing observation, resulting in one complete dataset that was exported to IMB SPSS Statistics version 30 for further analyses. All analyses were performed in SPSS version 30. Bivariate correlation analyses with the imputed data were conducted to examine associations between fathers’ testosterone levels and both CPB and protective parenting behaviors. We controlled for experimental condition in analyses involving protective parenting behavior.

## 3 Results

### 3.1 Correlations Between Fathers’ Hair Testosterone and CPB and Protective Behavior

Table 1 reports the descriptive statistics and Pearson correlations for the main variables of interest in both the complete and imputed dataset. Results showed no significant correlation between fathers’ hair testosterone levels and CPB without toys (*r*(70) = 0.01, *p* = 0.931) or CPB with toys (*r*(70) = 0.12, *p* = 0.307). The correlation between fathers’ hair testosterone levels and protective behavior was also nonsignificant, *r*(70) = 0.02, *p* = 0.894. The correlation between CPB without toys and protective behavior was nonsignificant (*r*(70) = 0.05, *p* = 0.701) as was the correlation between CPB with toys and protective behavior (*r*(70) = 0.18, *p* = 0.149).

**TABLE 1:** Descriptive statistics and Pearson correlations for the main variables based on complete data and imputed data.

Variable	*M* (SD)	Min–max	1	2	3	4	5
(1) Fathers’ hair testosterone levels	0 (0.21)	−0.44 to 70	**—**	0.02	0.15	−0.03	−0.32*
(2) CPB *without* toys	2.79 (0.58)	1.30–4.10	0.01	**—**	0.23	0.07	−0.16
(3) CPB *with* toys	2.10 (0.50)	1.30–3.40	0.12	0.22	**—**	0.18	−0.04
(4) Protective parenting	5.53 (1.92)	2–10	0.02	0.05	0.18	**—**	0.23
(5) Parental sensitivity	5.26 (1.41)	1.25–8.25	−0.30*	−0.16	−0.04	0.23	**—**

*Note:* Correlations based on complete cases are presented above the diagonal and pooled correlations for the 50 imputed datasets are presented below the diagonal. Hair testosterone concentrations are reported in pg/mL. Hair testosterone values were corrected for hair washed in the past 24 h via residualizing. Significant correlations are presented in bold.

*p < 0.05.

### 3.2 Exploratory Analyses (Not Preregistered)

Because parental sensitivity is of major importance for child development (Cooke et al. 2022; Madigan et al. 2024; Rodrigues et al. 2021), we conducted post hoc analyses to explore associations of paternal sensitivity with fathers’ hair testosterone levels, CPB, and protective parenting. These analyses were not preregistered and results should therefore be interpreted with caution. We found a significant negative correlation between fathers’ hair testosterone levels and paternal sensitivity, *r*(70) = −0.30, *p* = 0.013, indicating that fathers with higher testosterone levels engaged in less sensitive parenting during interactions with their 2‐ to 12‐month‐old infants. The effect size in the association between paternal sensitivity and protective parenting was substantial but fell just short of significance, *r*(70) = 0.23, *p* = 0.053. Correlations between paternal sensitivity and CPB with and without toys were nonsignificant. Exploratory analyses controlling for paternal sensitivity in the associations between fathers’ hair testosterone levels, CPB, and protective parenting did not alter the interpretation of the results. Furthermore, controlling for research visit (1, 2, or 3) in the associations between fathers’ hair testosterone levels and CPB did not affect the results.

Upon reviewer request, we repeated the analyses using multiple imputation procedures in SPSS. Fifty imputed datasets were generated with 20 iterations per imputation model. Correlation coefficients were pooled following Rubin's Rules. This did not change the results.

## 4 Discussion

The aim of this preregistered study was to examine associations between fathers’ hair testosterone levels and CPB and protective behavior. We hypothesized that fathers with higher hair testosterone levels would show more CPB and more protective parenting behaviors. Additionally, we expected that fathers who showed more CPB would engage in less protective parenting behaviors. In contrast to our hypotheses, we found no significant associations between fathers’ hair testosterone levels and CPB or protective parenting behavior. Furthermore, results indicated no significant associations between CPB and protective parenting behavior. Exploratory analyses indicated that fathers with higher hair testosterone levels were less sensitive during interactions with their infants.

There are various possible explanations for our null findings regarding the associations between fathers’ testosterone levels and CPB and protective parenting, some substantive and others methodological. First, fathers’ testosterone levels as obtained from hair may not actually be associated with their CPB and protective parenting behaviors. Hair testosterone levels reflect average testosterone concentrations over the past month and may be less affected by environmental and situational factors than saliva samples (De Vries et al. 2019; Grotzinger et al. 2018). Hence, hair samples might provide a more precise estimate of fathers’ basal testosterone levels and thereby allow for a more accurate estimation of their associations with CPB and protective parenting. Second, the temporal difference between the hair testosterone assessment and the observational measures of CPB and protective parenting behavior may have obscured potential associations. Protective behaviors and CPB may be more situationally variable than behaviors such as paternal sensitivity, reducing the likelihood that a single observation provides a representative picture. However, research showed that CPB seems to have some modest cross‐situational and cross‐temporal stability (Majdandžić et al. 2016). Future research may benefit from assessing protective parenting behaviors and CPB across multiple timepoints to ensure greater temporal correspondence between hormonal and behavioral assessments. Relatedly, it may be that diurnal patterns or situational fluctuations in testosterone levels are more strongly related to momentary CPB and protective parenting behavior than testosterone levels measured in hair. A third explanation for the nonsignificant associations may be that this study was sufficiently powered to detect medium‐sized effects, but not lower effects sizes. In the Meijer et al. (2019) meta‐analysis, the reported effect size between fathers’ testosterone levels and parenting practices was small (*r* = 0.07, *k* = 0 18). Small associations between fathers’ hair testosterone levels and CPB and protective parenting behavior may have gone undetected because the study was not sufficiently powered to detect small effects. Of note, most studies in the meta‐analysis used salivary assessments of testosterone. Fourth, we focused on one hormone. Research showed that among fathers with higher testosterone levels, oxytocin was negatively associated with affectionate touch (Gordon et al. 2017). Another study found that oxytocin administration increased fathers’ testosterone levels, and that this change in testosterone levels was associated with fathers’ behavior, including expressions of positive arousal, social gaze, and vocal synchrony. (Weisman et al. 2014). Additionally, studies demonstrated that interactions between testosterone and estradiol (Bakermans‐Kranenburg et al. 2022) and between testosterone and cortisol predicted paternal sensitivity (Lotz et al. 2022). Speculatively, the interplay between testosterone and other hormones may better predict CPB and protective parenting behavior, although this suggestion warrants further investigation.

Of note, as argued by Lotz et al. (2021), the observational task used to measure fathers’ protective behavior primarily captures protective responses directed toward the child, such as covering the infant's ears, rather than protective aggression directed toward the source of the threat. Future studies may examine whether fathers’ testosterone levels are differently related to protective caregiving and protective aggression. Another point to raise with regard to the association between fathers’ hair testosterone levels and CPB is that the children in this sample were between 2 and 12 months old. A review of the literature presented some support for the idea that fathers engage in more activation parenting, including CPB, after their children's first birthday (J. Feldman and Shaw 2021). It is currently unknown whether associations between fathers’ hair testosterone levels and CPB emerge in samples of fathers with older children.

We found no significant association between CPB and protective parenting behavior, whereas previous research found significantly negative associations when assessing these behaviors in the same contexts (Majdandžić et al. 2016). We observed these behaviors during two different tasks: CPB was observed during a free play interaction and protective parenting behavior was observed in response to exposure to a loud sound, that is, the AST. An explanation for the absence of an association may be that fathers adapt their behavioral responses to contextual demands. A father who shows CPB in a safe and playful context may or may not be protective in a threatening context.

It has been argued that a decrease in testosterone levels reflects a shift from pursuing mating opportunities toward greater investment in offspring and the couple relationship (Gettler et al. 2014; Gray and Campbell 2009; Wingfield et al. 1990). In line, results from our exploratory analyses suggest that lower testosterone levels may pave the way for more sensitive parenting. These findings add to the mixed results in the literature about the association between testosterone and sensitive and nurturing behaviors (Bakermans‐Kranenburg et al. 2022; Bos et al. 2018; Endendijk et al. 2016; Lotz et al. 2022) and are in line with meta‐analytic evidence showing that lower testosterone levels are related to higher parenting quality (Meijer et al. 2019). Likewise, another study reported that fathers with lower testosterone levels engaged in more positive parenting and less negative parenting during interactions with their 18‐month‐old children (Rodriguez et al. 2021). Although our findings similarly point to a direct association between fathers’ testosterone levels and paternal sensitivity in infancy, replication studies are needed. Notably, our research design precludes definitive conclusions about the direction of this association.

Strengths of this study include its preregistration, hair samples instead of saliva samples as a measure of testosterone, and the use of observations to measure CPB and protective parenting in a semi‐naturalistic laboratory setting. Yet, there are also several limitations that should be considered when interpreting our findings. First, the sample consisted of first‐time partnered fathers who were predominantly highly educated and from Dutch origin. It remains unknown whether findings can be generalized to samples with different backgrounds and nationalities. Second, missing values were imputed to reduce data loss and potential bias, but the relatively high number of missing testosterone data may have affected the precision of the observed associations between testosterone, CPB, and protective parenting behavior. However, complete cases showed similar results compared to the imputed data set. Third, we focused on fathers’ protective behavior in response to their infant's exposure to a loud sound, which may represent an emotional or sensory threat. Future research may examine whether fathers’ hair testosterone levels are more strongly associated with protective behavior in situations involving physical or social threats, such as when a child is in danger or excluded by others. This may be assessed using virtual reality paradigms that can safely simulate these types of threats.

In sum, this was the first study to examine associations between fathers’ hair testosterone levels, CPB, and protective parenting behavior toward their infant. We found no significant correlations between any of these measures. Future studies are needed to examine whether associations emerge in larger samples, ideally including fathers with more diverse backgrounds and fathers of older children. Furthermore, future studies may benefit from incorporating multiple testosterone indices (i.e., hair, diurnal, and reactivity measures) to examine whether these indices are differentially related to CPB and protective parenting behavior. This will provide a more nuanced understanding of the relationships between testosterone, CPB, and protective parenting. Exploratory analyses showed that fathers with lower testosterone levels were more sensitive during interactions with their infants. These findings add to similar results in the literature and may help to better understand the hormonal mechanisms underlying paternal sensitivity.

## Author Contributions

**Annemieke M. Witte**: methodology, formal analysis, investigation, data curation, writing – original draft, visualization, project administration. **Carolina Santos**: methodology, writing – review and editing. **Mirjana Majdandžić**: investigation, resources, writing – review and editing. **Marinus H. van IJzendoorn**: conceptualization, writing – review and editing, funding acquisition. **Marian J. Bakermans‐Kranenburg**: conceptualization, investigation, writing – review and editing, supervision, funding acquisition

## Funding

This research was supported by a European Research Council grant (ERC AdG 669249) awarded to Marian J. Bakermans‐Kranenburg and a NWO Spinoza prize awarded to Marinus H. van IJzendoorn.

## Conflicts of Interest

The authors declare no conflicts of interest.

## Supporting information




**Supplementary figure**: dev70160‐sup‐0001‐FigureS1.docx

## Data Availability

The data that support the findings of this study are available from the corresponding author upon reasonable request
