# Supplementary material for: Testosterone Levels, Challenging Parenting Behavior, and Protective Parenting Behavior: A Correlational Study Among First‐Time Fathers in the First Year of Fatherhood
Source: Dev Psychobiol. 2026 May 8;68:e70160. doi: 10.1002/dev.70160 (PMC13155649; doi:10.1002/dev.70160)
Supplement: Supplementary file 1 — Supplementary figure: dev70160‐sup‐0001‐FigureS1.docx [file DEV-68-e70160-s001.docx]

**Supplementary Materials**

**Figure 1**. Reasons for missing data

Missing data due to:

- Insufficient hair material (*n* = 12; testosterone)
- Hormonal levels undetectable (*n* = 12; testosterone)
- Baldness (*n* = 3; testosterone)
- COVID-19 (*n* = 5; CPB, paternal sensitivity; *n* = 4/5 also protective parenting)
- COVID-19 (*n* = 2; protective parenting)
- Drop-out (*n* = 4; CPB, paternal sensitivity, protective parenting)
- Infant not clearly visible (*n* = 1; paternal sensitivity)
- Speaking different language with infant (*n* = 1; verbal CPB*).
- Technical issues (*n* = 4; protective parenting)
- Researcher entering the room (*n* = 1; protective parenting)

*Note*. CPB = Challenging Parenting Behavior. * For this participant, only the physical CPB scale scores were included in the scores for CPB *without* toys and for CPB *with* toys.
